# Toxicity by descent: A comparative approach for chemical hazard assessment

**DOI:** 10.1016/j.envadv.2022.100287

**Published:** 2022-10-01

**Authors:** John K. Colbourne, Joseph R. Shaw, Elena Sostare, Claudia Rivetti, Romain Derelle, Rosemary Barnett, Bruno Campos, Carlie LaLone, Mark R. Viant, Geoff Hodges

**Affiliations:** a Michabo Health Science Ltd, Coventry CV1 2NT, UK; b School of Biosciences, University of Birmingham, Edgbaston B15 2TT, UK; c O’Neill School of Public and Environmental Affairs, Indiana University, Bloomington 47405, USA; d Safety and Environmental Assurance Centre, Unilever, Colworth Science Park, Sharnbrook MK44 1LQ, UK; e US Environmental Protection Agency, Duluth 55804, USA

**Keywords:** Evolutionary toxicology, Cross-species extrapolation, Genomics, Model species, Comparative toxicology, Biomarkers

## Abstract

Toxicology is traditionally divided between human and eco-toxicology. In the shared pursuit of environmental health, this separation does not account for discoveries made in the comparative studies of animal genomes. Here, we provide evidence on the feasibility of understanding the health impact of chemicals on all animals, including ecological keystone species and humans, based on a significant number of conserved genes and their functional associations to health-related outcomes across much of animal diversity. We test four conditions to understand the value of comparative genomics data to inform mechanism-based human and environmental hazard assessment: *(1)* genes that are most fundamental for health evolved early during animal evolution; *(2)* the molecular functions of pathways are better conserved among distantly related species than the individual genes that are members of these pathways; *(3)* the most conserved pathways among animals are those that cause adverse health outcomes when disrupted; *(4)* gene sets that serve as molecular signatures of biological processes or disease-states are largely enriched by evolutionarily conserved genes across the animal phylogeny. The concept of homology is applied in a comparative analysis of gene families and pathways among invertebrate and vertebrate species compared with humans. Results show that over 70% of gene families associated with disease are shared among the greatest variety of animal species through evolution. Pathway conservation between invertebrates and humans is based on the degree of conservation within vertebrates and the number of interacting genes within the human network. Human gene sets that already serve as biomarkers are enriched by evolutionarily conserved genes across the animal phylogeny. By implementing a comparative method for chemical hazard assessment, human and eco-toxicology converge towards a more holistic and mechanistic understanding of toxicity disrupting biological processes that are important for health and shared among animals (including humans).

## 1. Introduction

Although vertebrates including mammals such as mice and rats were historically considered the ‘gold standard’ in regulatory toxicity testing of industrial chemicals, ample evidence demonstrates that these animals are not consistently reliable predictors of chemical harm in humans (Bailey et al., 2014). This limitation, coupled with the desire to improve throughput and reduce experimentation on animals, has driven recent toxicology efforts to instead focus on non-animal approaches for use in chemical safety assessment. Examples of such approaches include *in vitro* testing using human-derived cell lines (Ghallab and Bolt, 2014), the derivation of points of departure (POD) from *in vitro* bioactivity data (Paul-Friedman et al., 2020), and the use of transcriptomics and metabolomics (Harrill et al., 2021; Viant et al., 2019) with a focus on applying a mechanistic understanding of toxicology to the protection of human health. Similarly, recent efforts to predict developmental toxicity based on changes in cellular metabolism are providing useful results for inclusion into human health hazard assessments (Zurlinden et al., 2020).

These advancements in using non-animal approaches to assure safety have also encouraged efforts to better understand the shared molecular targets and pathway conservation across species to mutually inform both human and environmental health (Rivetti et al., 2020). Strides to merge existing knowledge of biological pathways across species in a well-defined and consistent framework, with an evaluation of the weight of evidence, are defined as the adverse outcome pathway (AOP) (Ankley et al., 2010; Vinken, 2013). The AOP framework focuses on understanding the conservation of toxicity pathways across the different levels of biological organization to link molecular level changes, which can be measured in high-throughput bioassays or omics approaches, to apical individual or population level adverse outcomes relevant to risk assessment, such as cancer or reproductive abnormalities (Brockmeier et al., 2017). By capturing existing pathway knowledge to inform risk assessment from all species, it becomes clear that diverse species share similar biology, which should provide valuable mechanistic information relevant to both human and environmental risk assessment.

Historically, biomedical researchers have relied on a diverse suite of model organisms that includes invertebrates such as *Drosophila melanogaster* (an insect) and *Caenorhabditis elegans* (a nematode) to understand basic biology and the human condition (Bhattacharjee et al., 2019; Fields and Johnston, 2005; Hedges, 2002; Muller and Grossniklaus, 2010) with great effect at discovering a more complete spectrum of the genetic causes of human illness, including neurodegenerative disorders, cancer, cardiovascular kidney and hepatic diseases (Bonini and Berger, 2017; Cacheiro et al., 2019; Perveen, 2018; Potter et al., 2000; Shin and Fishman, 2002; Wilson-Sanders, 2011). Invertebrates such as *Drosophila* and *Caenorhabditis* are chosen for their genetic and molecular tractability and used as resource materials for exhaustive investigations of processes that are fundamental in biology and shared by other species, especially with humans (Ankeny and Leonelli, 2011). By contrast, the historic use of two distinct sets of animal models for toxicology emphasizes separate practices for assessing health risks. Mammals or other vertebrates have been historically used as human health surrogates, while ecologically relevant species (such as a fish, a cladoceran zooplankton and an algal species representing the different trophic levels of an aquatic food chain) have been used as sentinels of environmental health (National Research Council, 2014; Gad, 1990). This difference in the use of surrogate species has meant that, whilst biomedical science has rallied around the diversity of its models by adopting the concept of homology (Institute for Laboratory Animal Research U.S. Committee on New and Emerging Models in Biomedical and Behavioral Research 1998), the toxicology community has been slow to put into practice the greater use of knowledge from diverse species to understand chemical hazards.

The concept of homology applied in biomedical research is a product of comparative biology (Hall, 1994); the experimental observations from one species to another are made under the testable hypothesis that their similarities are due to an evolutionary history written within their genomes (Brigandt, 2003). Therefore, unlike in most of toxicology, the rules of correspondence between species are based on a significant body of knowledge from evolutionary genomics that serves as a guide to establishing appropriate criteria for identifying molecular processes (including toxicity) that are most likely to be shared between animals by common ancestry.

Generally, the species that diverged most recently share the greatest number of genes. For example, only around 100 genes are unique to humans (1%) when compared to other primates (Zhou et al., 2014), while only around 15% of human genes share a common descendant with *Drosophila* genes (Shih et al., 2015). Yet, despite divergence times greater than 780 million years since insects and mammals last shared a common ancestor (Hedges et al., 2006), over 65% of human disease-causing genes may have functional homologs in flies, based on an early comparison of the two genomes (Reiter et al., 2001; Yamamoto et al., 2014). Broader taxonomic surveys also suggest that most disease-causing genes are also the most ancestral genes of animal genomes (Domazet-Loso and Tautz, 2008; Forslund et al., 2011; Hwang et al., 2014; Maxwell et al., 2014; O’Brien et al., 2004; Rubin et al., 2000). For example, by tracing the last common ancestor in which a human gene exists, Domazet-Loso and Tautz (2008) demonstrated that most disease genes predate animal evolution. By having access to a broader phylogenetic range of animal genomes, Maxwell et al. (2014) later demonstrated that not every class of human disease genes is ancient, specifically pointing to inflammatory and immunological diseases as having large proportions of vertebrate-specific genes. These and other studies are reinvigorating comparative studies across a greater diversity of experimental model species to understand the human condition (Gerstein et al., 2014). For toxicology, such comparative phylogenetic methods at identifying deep homologies for functional elements of genomes and their associations among biomedical model species offer a useful roadmap to ultimately identify toxicological homologies (toxicity by descent) and inform both human *and* environmental health.

For a start, we test four conditions that should accelerate the use of biomarkers in non-vertebrate toxicity testing, demonstrating to what degree human genes and their functional associations into pathways that, when perturbed, are involved in disease are comparatively conserved among vertebrate and invertebrate genomes. This work is motivated by growing epidemiological evidence that disease is often-times a result of an interaction of genes and environmental conditions, including exposure to pollutants (Mbemi et al., 2020), and is pursued by mining peer-reviewed databases of homologous genes and pathways (Fabregat et al., 2018; Kriventseva et al., 2019).

Condition 1 – Vertebrate model species have more genes in common with humans than invertebrate genomes (Prachumwat and Li, 2008). Yet the phylogenetic distribution of disease genes across species is significantly larger than genes not involved in disease. Such a finding would reinforce observations made in other fields of research (Domazet-Loso and Tautz, 2008; Lopez-Bigas and Ouzounis, 2004); biological processes and their genes that are most fundamental for health have deeper evolutionary origins in the animal phylogeny. Therefore, data historically generated in non-mammalian or non-vertebrate species can be used in a weight-of-evidence approach and provide mechanistic information relevant to both human and environmental hazard assessment.

Condition 2 – Connected molecular events (i.e., reactions within pathways) are better conserved among distantly related species than the individual genes that are members of these pathways (Gerstein et al., 2014; Stuart et al., 2003). Such a finding would support a growing interest among toxicologists in adverse outcome pathways (AOPs), which describe apical target activation and the molecular key events leading to toxicity based on chemical modes of action (Sturla et al., 2014; Waters and Fostel, 2004). By emphasizing the discovery of evolutionarily conserved pathways that are susceptible to chemical interference leading to harm (Barabasi et al., 2011; de la Fuente, 2010), a greater diversity of test species can be used in helping to understand mechanisms of toxicity.

Condition 3 – The most conserved pathways among animals are those that, when perturbed, cause adverse outcomes including disease. This third condition is a logical extension of the prior two, testing whether a relatively earlier ancestry of genes (condition 1) and their functional interactions (condition 2) fundamental for health also apply to their canonical pathways. Such a finding would encourage the environmental health research community to fill a knowledge gap to prevent exposure-related harm – by building upon the already huge investments in the discovery and classification of disease pathways, most of which have unknown associations with environmental conditions including exposure to toxicants.

Condition 4 – Molecular signatures databases (gene sets; (Kramer et al., 2014; Liberzon et al., 2015)) that provide medicine with new predictors (i.e., biomarkers) of specific biological states or disease processes can be used for hazard detection in toxicology. A finding that these gene sets are largely enriched by evolutionarily conserved genes across the animal phylogeny, would further demonstrate the feasibility of classifying chemicals for their harmful effects on animals including humans, based on gene expression monitoring of genes (and their pathways) already serving as biomarkers, using a small suite of model test species.

Because genes that are associated with disease are not necessarily those that result in toxicity from chemicals, we conclude by evaluating the likelihood that existing biomarkers of chemical exposure (the US National Toxicology Program’s s1500+ reference gene panel) that are specific to human xenobiotic and stress response pathways may be indicative of exposure-related health outcomes using distantly related test species. The overall objective of this study is to investigate the degree to which biomolecular pathways that are relevant for assessing chemical hazards to humans can be informed by the study of alternative (non-vertebrate) model test species.

## 2. Methods

Most molecular genetic knowledge on the function of human genes derives from experiments that have been performed using other species (Ankeny and Leonelli, 2011). This success at extrapolating a gene’s function from one organism to another largely depends upon the degree by which natural selection (evolution) acts at preserving the gene – starting at its origin from a common ancestor in the animal phylogeny, then throughout the speciation events that delineate the assemblages of animals being compared. For example, if a gene is preserved in the genomes of all species that are descendants of a common ancestor that had evolved the gene, with few other copies created by gene duplication, then there is a high probability that its homologs have the same function in other species (Gabaldon and Koonin, 2013; Rogozin et al., 2014). Such homologous genes that are shared by descent, because of speciation events only, are defined as *orthologs* (Koonin, 2005). However, if the evolution of such genes also involves many gene duplications or gene deletions within the genomes of species, then it is generally predicted that these homologs may have modified the original gene function (Guschanski et al., 2017). Such homologous genes that are shared by descent, yet have additional copies that were acquired after speciation events, are defined as *paralogs* (Koonin, 2005). Altogether, orthologs and paralogs form *gene families* that are distributed differently among species. Here for our investigations, we assume that the functions of the progenitor gene are retained by at least one member of the gene family when present within species.

Given the fundamental importance of tracing the genealogy of genes for effective cross-species extrapolation of their biological functions, there are over a dozen active projects to create ever more reliable gene families (Linard et al., 2021). For this study, we data mined the ortholog resource OrthoDB (Kriventseva et al., 2019) to test Condition 1 by investigating the species distribution of homologous protein-coding gene families across a suite of 12 species (*Suite 1*) that are relevant to human and eco-toxicology (Table 1). By contrast, our investigation of homologous pathways to test Conditions 2 and 3 among five vertebrates and two invertebrates (*Suite 2*; Table 1) was based on biological pathway information provided by the Reactome project (Fabregat et al., 2018). Here, pathways are composed of genes participating in reactions, thereby forming distinct networks of biological interactions. Reactions are defined as any event that transforms a biological molecule. The orthology of pathway interacting genes across species is also based on phylogenetic trees, using the Ensembl Compara resource (Herrero et al., 2016) instead of OrthoDB. All comparisons among species were made concerning humans, both for gene families and homolog pathways. In this respect, we intentionally ignored lineage-specific genes and pathways that are shared among species excluding *Homo sapiens*. Therefore, we focus our study on methods by which hazards to humans can be informed by distantly related test organisms, such as invertebrates.

### 2.1. Condition 1: relative conservation of disease and non-disease genes

The presence and absence of annotated human protein-coding genes within genomes of the 11 other animals (Suite 1) were obtained by querying OrthoDB (release version 10) using SPARQL. To improve consistency among the species annotations, only genes that are assigned NCBI gene IDs (Sayers et al., 2019) were counted as members of gene families, save for the *Hyalella* genes that required Uniprot gene IDs (Bateman et al., 2019). A total of 10,441 gene families shared genes between humans and at least one other vertebrate that was included in this study at the lowest common ancestor level (i.e., Bilateria). The final count of homologous genes within this study is 155,962 – of which 18, 517 belong to the human genome, representing 84% of all human protein-coding genes.

The gene families were classified as “disease” if all paralogs from the human genome were linked to disease outcomes obtained from the OMIM Morbid Map generated on 20/12/2018 (Amberger et al., 2019). The OMIM is a continuously updated authoritative compendium of human genes and genetic disease phenotypes. Alternatively, the gene families were classified as “non-disease” or “mixed” if none or some human genes were linked to disease outcomes respectively. A total of 1597 gene families were thus classified as “disease” while 7446 were classified as “non-disease”. The remaining 1398 “mixed” gene families were excluded from our investigation thereby comparing only two classes of genes.

To test whether genes influencing human health (“disease” class) have an evolutionary history that is different from genes that are unassociated with disease (“non-disease” class), we transformed the numerical profiles of their gene families into binary profiles. The evolutionary origins of these two gene classes were then inferred by “reconstructing” the distribution of these gene families among ancestral species (the internal nodes of the animal phylogeny) based on their presence or absence among the twelve extant species (the external branches of the tree) by a method called Dollo parsimony (Rogozin, 2006). In maximum parsimony, the best evolutionary explanations for the distribution of characters among species are those that minimize the number of evolutionary steps of these characters (i.e., gain and losses). Yet, under Dollo’s law of irreversibility, parsimony is constrained, whereby a character appears only once and can never be regained if it is lost (i.e., no convergence nor reversions). This analysis was performed using the Count software package (Csuros, 2010). The significance of our findings was tested using the z-score, by independently comparing the number of disease gene gains and losses at each branch of the phylogeny against the mean numbers and distributions from 1000 random sub-samplings of the non-disease genes.

### 2.2. Condition 2: relative conservation of reactions within pathways

The homologous pathways shared between humans and six other species (Suite 2) were obtained by querying the Reactome database (version 67 on 25/01/2019), and by recording their number of gene families and their number of conserved reactions for each species with reference to *Homo,* using the embedded “species comparison” tool. To ensure that summaries of our results consisted of independent non-nested gene networks, only the terminal pathways (lowest level, with no dependents) were included in this study. This condition reduced the number of human pathways from 2180 to 1773. On average, these pathways contained 29 genes with UniProt assignments and 11 reactions. We finally excluded from our investigation 264 pathways that had fewer than three elements and one generic transcription pathway that contained 1228 elements, resulting in a final set of 1508 gene pathways.

Frequency distributions for the average percentage of conserved ortholog group and reactions within pathways were compared for both the non-human vertebrates and the invertebrates using JMP Pro (JMP^®^, Version 14.1. SAS Institute Inc., Cary, NC, 1989–2019). The following generalized linear model (GLM) was applied to investigate whether the conservation of human pathways within invertebrates could be predicted by the level of pathway conservation in the more closely related non-human vertebrates:

Pathway conservation among invertebrates ~ (average vertebrate conservation) + (# genes in *Homo* pathway) + (average vertebrate conservation : # genes in *Homo* pathway).

### 2.3. Condition 3: relative conservation of disease and non-disease pathways

Just as we compared the relative conservation of disease and non-disease-associated genes, we also compared the relative conservation of disease and non-disease-associated pathways (Suite 2); of the 1508 gene pathways under investigation, 351 were annotated by the Reactome Project as being associated with disease. We used a phylogeny-based maximum likelihood inference model to determine the evolutionary rates at which genes and reactions were lost along each branch of the phylogenetic tree (Csuros, 2010). This method differed substantially from maximum parsimony because we wished to preserve the information on the relative number of shared genes among species for all networks (not to transform the numerical profiles to presence and absence of pathways) while using a statistical approach. Yet, by setting the appropriate model parameters to account for losses and not for gains or duplications, this analysis equally obeyed Dollo’s law of irreversibility. As a result, a statistical phylogenetic estimation was obtained, on *when* elements (i.e., gene families) of the networks were likely lost along the animal phylogeny by computing likelihoods and posterior probabilities for ancestral network content. This analysis was performed using the Count software package (Csuros, 2010) by first optimizing the evolutionary rates for all branches of the phylogeny using a “pure loss” model and by setting the network size to be Poisson-distributed at the root of the tree. The numbers of pathways that were altogether lost or had contracted to only a single ortholog group or reaction were then calculated from their posterior distributions at each branch.

### 2.4. Condition 4: conservation of hallmark signatures of potential adverse outcome pathways

The Molecular Signatures Database (MSigDB; (Liberzon et al., 2015)) is a collection of annotated gene sets that are used in biomedical research and drug development as indicative biomarkers of biological processes and disease pathologies. These gene sets are divided into 8 major collections for use in a gene set enrichment analysis (GSEA; (Subramanian et al., 2005)). The 50 hallmark gene sets we used in our analysis were computationally generated at the Broad Institute of MIT and Harvard (Cambridge, U.S.A) as displaying coordinated expression (Liberzon et al., 2015). To apply these to our study, we simply exchanged the human gene IDs within all gene sets with the ortholog group ID of their gene families while retaining only unique entries. To then perform a GSEA, we explored the applicability of the Shannon index in measuring the distribution of genes (Spellerberg and Fedor, 2003), to rank order the 10,441 gene families, based on their distribution across 12 animal species (i.e., their conservation in Suite 1). Therefore, the two required input files for the GSEA pre-ranked tool consisted of a “hallmark ortholog set” and a list of gene families sorted in descending order, based on the Shannon index. This “ortholog set enrichment analysis” was performed using the JAVA desktop GSEA application (Subramanian et al., 2005) and repeated for 1266 ortholog sets that met the GSEA set size criteria (min=3, max=500) from among the 1508 Reactome pathways. Furthermore, we tested the potential utility of the comparative approach for regulatory toxicology by repeating this ortholog set enrichment analysis on the S1500+ biomarker panel, which consists of 2753 human genes chosen to represent pathways that are relevant to the United States National Toxicology Program (NTP). Although 1600 genes from this NTP set overlap with either the hallmark gene sets (33%), disease genes (10%), or both (16%) (Supplemental Figure A1), a significantly higher degree of evolutionary conservation for the full NTP set of biomarker genes would further validate their functional association to critical biological processes, shared among distantly related species.

## 3. Results

### 3.1. Condition 1: relative conservation of disease and non-disease genes

A first test of a comparative approach for chemical hazard assessment is the conservation among species of human genomic elements influencing health. Because of growing evidence that key biological processes – which are fundamental to life – have early evolutionary origins and are thus governed by suites of genes that are widely shared among animal species (e.g., Seebacher, 2018), we deliberately test whether genes that are linked to human diseases are disproportionally represented in all animal genomes. To evaluate the conservation of human genes linked to disease, relative to those that are unassociated with disease, we identified the human orthologs in the genomes of 11 other species for 10,012 loci listed within the OMIM database (Supplemental Tables A1–A3). Of the 1597 human disease gene families, 414 (26%) have homologs among all species. By contrast, of the 7446 non-disease gene families, only 1327 (18%) have homologs among all species (Table 2). The remaining 1398 gene families are composed of genes from both disease classes. Despite this significant difference (p < 0.01), both classes share a similar proportion of single-copy gene families (40% *vs* 41%), which are those genes that have not been duplicated throughout animal evolution. As expected because of a more recent ancestry, a greater proportion of genes encoded in the human genome are shared with another vertebrate, for both disease (99.9%) and non-disease (99.8%) classes. This observed difference between the conservation of both gene classes (0.1%) is larger (11.9%) for genes that are also present within an invertebrate genome (70.9% *vs* 59.0%; Table 2). Therefore, although only 64% of gene families of vertebrate genomes are overall shared with invertebrate genomes (6671 of 10,441 gene families), this fraction is significantly enriched by genes of the human genome influencing health (*p* < 0.01).

**Fig. 1.**
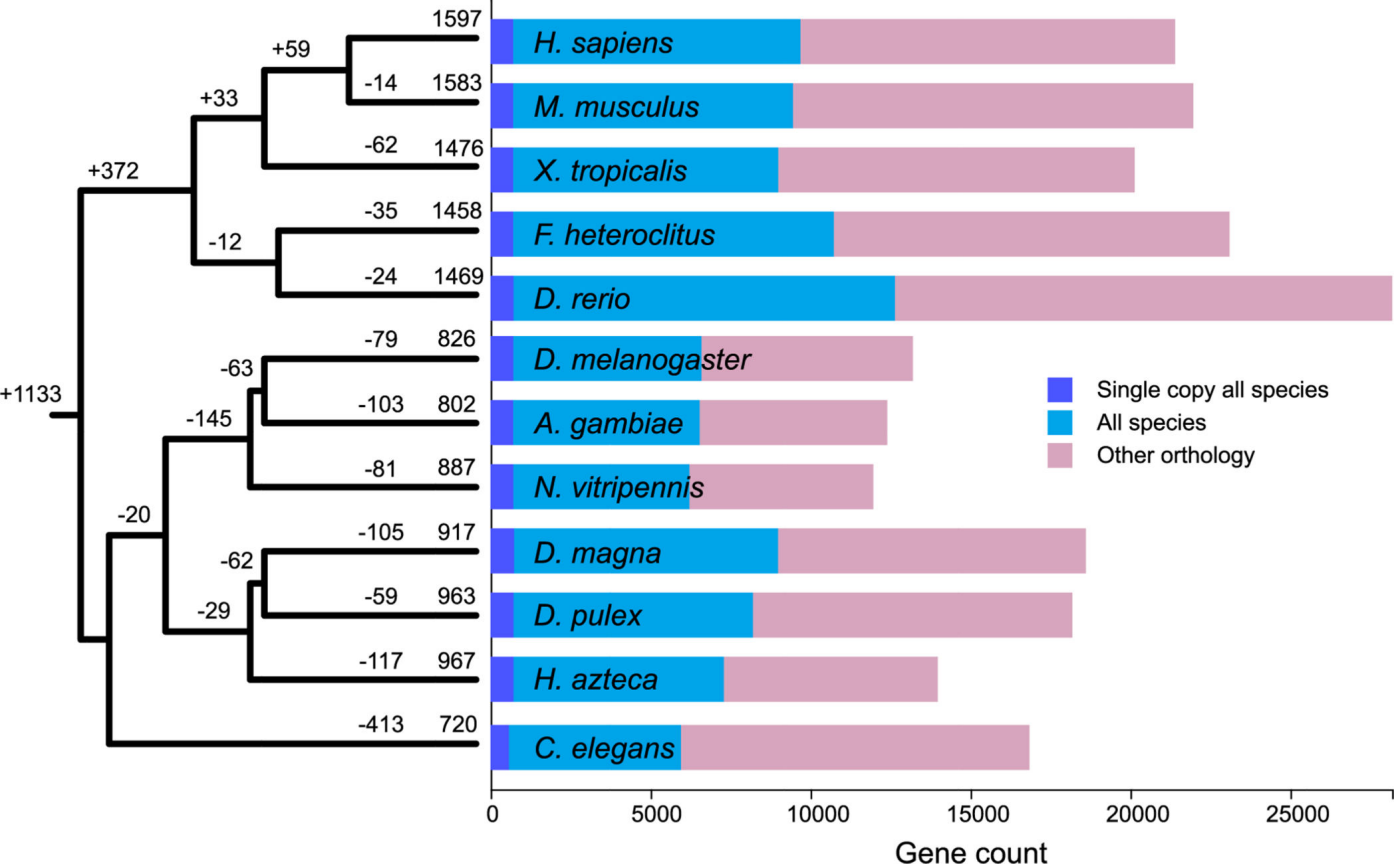
Reconstruction of the origins of disease-associated gene families by Dollo parsimony, mapped onto a skeletal animal phylogenetic tree representing mammals (*Homo, Mus*), tetrapods (mammals plus *Xenopus*), fish (*Danio, Fundulus*), vertebrates (tetrapod plus fish), insects (*Drosophila, Anopheles, Nasonia*), crustacea (*Daphnia, Hyalella*), arthropods (insects plus crustacea) and ecdysozoa (arthropods plus *Caenorhabditis*). The gene counts are transformed into binary (presence/absence) profiles prior to mapping the number of gene family gains (+) and losses (−) for each branch of the tree. The overall number of orthologs that are shared among the 12 species are shown as a bar-chart.

Indeed, a phylogenetic reconstruction of the evolutionary origins of disease gene families indicates that 1133 (71% of total) originate at the root of the animal phylogeny (Fig. 1); only 372 such gene families (23% of total) uniquely originate in a vertebrate descendent. In other words, by assuming that gene families are unlikely to have originated independently more than once (an assumption called Dollo Parsimony; (Rogozin, 2006)), the ancestral genome of all animal species possessed most loci of interest for understanding human health. These were then subsequently and disproportionately lost along the three invertebrate lineages. For example, the diminutive *C. elegans* genome contains the smallest number of gene families that are shared with all other species, including the loss of 413 human disease gene families (Fig. 1). By contrast, the lineage leading to insects lost 145 human disease gene families, while only 29 are lost by the descendant of crustaceans. This finding suggests that, although insects such as *Drosophila, Anopheles* and (to a lesser extent) *Nasonia* are used to study the human condition (Werren et al., 2010), crustaceans currently serving as model test species in ecotoxicology are potentially better human surrogates for exposure-related hazards (Colbourne et al., 2011). For example, *D. magna* retains 917 disease causing gene families compared to 826 for *D. melanogaster* (Fig. 1). This finding reflects an overall greater preservation of ancestral gene families by crustaceans compared to insects (see gene count axis in Fig. 1). However, we discover that the preservation rates of disease gene families in the arthropods are barely greater than the preservation rates for non-disease-causing gene families (z-score <1) thereby suggesting that the earlier origin of disease-causing gene families at the root of the animal phylogeny is sufficient to explain their over-representation within invertebrate genomes (Table 3).

**Fig. 2.**
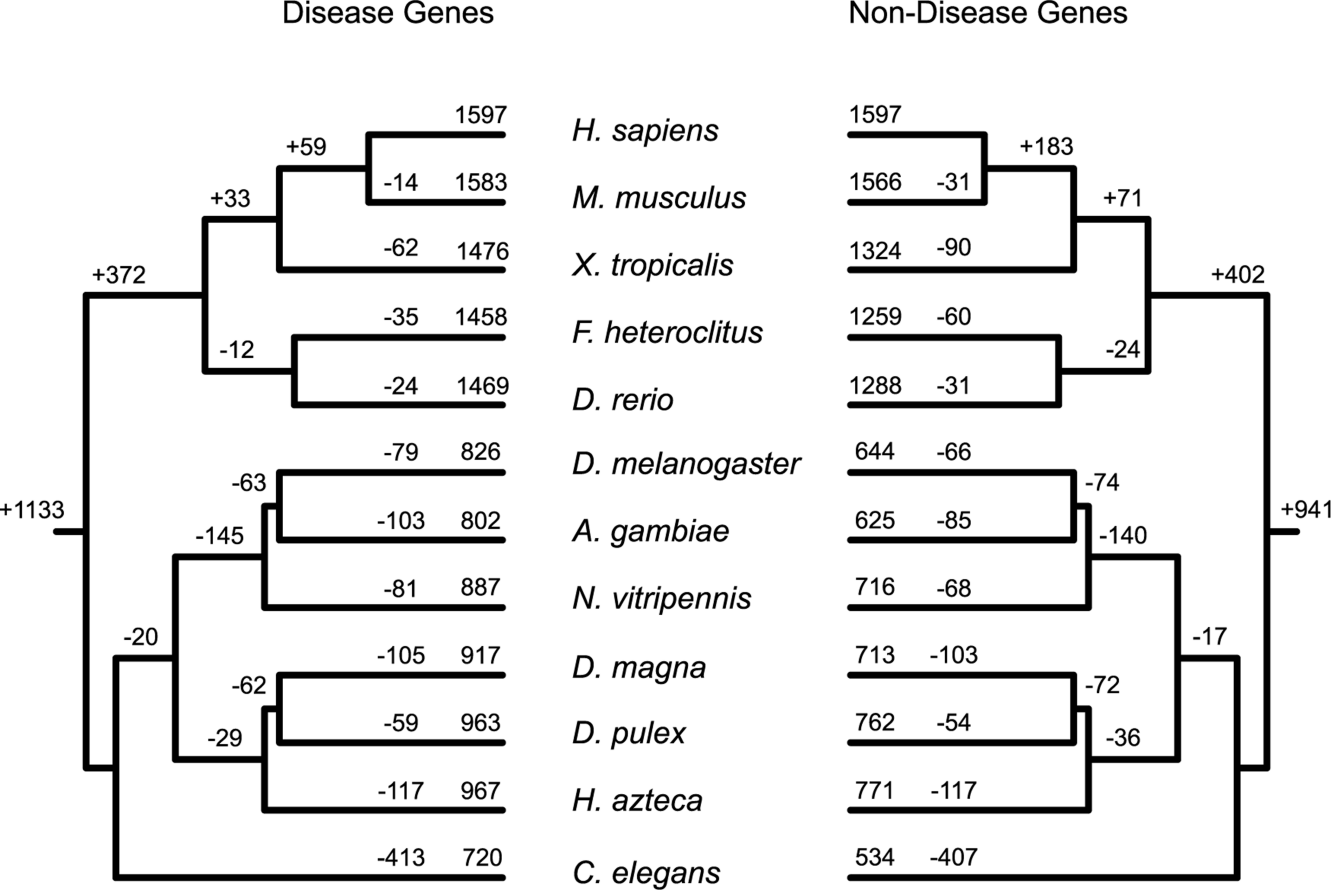
Comparison of the origins of both disease (left panel) and non-disease (right panel) associated gene families by Dollo parsimony. Although the number of disease gene families are actual, the number of non-disease-associated gene families are averages from 1000 sub-sampling of 7447 orthologous groups (see Table 3).

To better compare conservation rates between these two classes of gene families, a similar phylogenetic reconstruction of the likely evolutionary origins of non-disease genes indicates that 20% fewer gene families have origins at the root of the animal phylogenetic tree (average of 941 *versus* 1133; Fig. 2). Therefore, a significantly larger number of these gene families uniquely originate in a vertebrate, tetrapod and mammalian descendent. Yet despite significantly greater preservation rates of disease gene families compared to non-disease genes along all branches of the vertebrate phylogeny (Table 3), this pattern is not apparent for the descendant of the arthropods (20 *versus* and average of 17 gene family losses), nor of the insects (145 *versus* and average of 140 losses; Fig. 2).

Therefore, the prediction of this first test of the comparative approach holds true. Over 70% of gene families linked to disease evolved early in the animal phylogeny and were retained disproportionately among the invertebrate lineages. In other words, genes that are likely to be most informative at predicting adverse health outcomes are shared among the greatest number of species through evolution.

### 3.2. Condition 2: relative conservation of reactions within pathways

A second test of the comparative approach for chemical hazard assessment is the conservation of interactions among genes, which function within identifiable human pathways influencing health. Pathways are here defined as interacting genes of animal genomes that form gene networks performing a specific molecular function or a biological process. For example, by integrating results obtained by both the ENCODE and modENCODE projects (comparing the human, fly and nematode genomes; (Gerstein et al., 2014)) a significant suite of genes and their functional associations for growth, maintenance and reproduction are shared among animals – representing over 60% of transcribed and epigenetically modified genomes (Gerstein et al., 2014). Cumulating evidence suggests that functional dependencies among interacting genes forming networks can produce strong epistatic constraints on evolutionary processes that would otherwise create greater differences among species (Taute et al., 2014). We, therefore, test whether gene networks and their reactions are more conserved among distantly related species than the genes that form these networks. To evaluate the relative conservation of pathways between vertebrate and invertebrate species, we interrogated the Reactome pathway database for interacting genes and the reactions that are homologous across eight species (Supplemental Table A4).

**Fig. 3.**
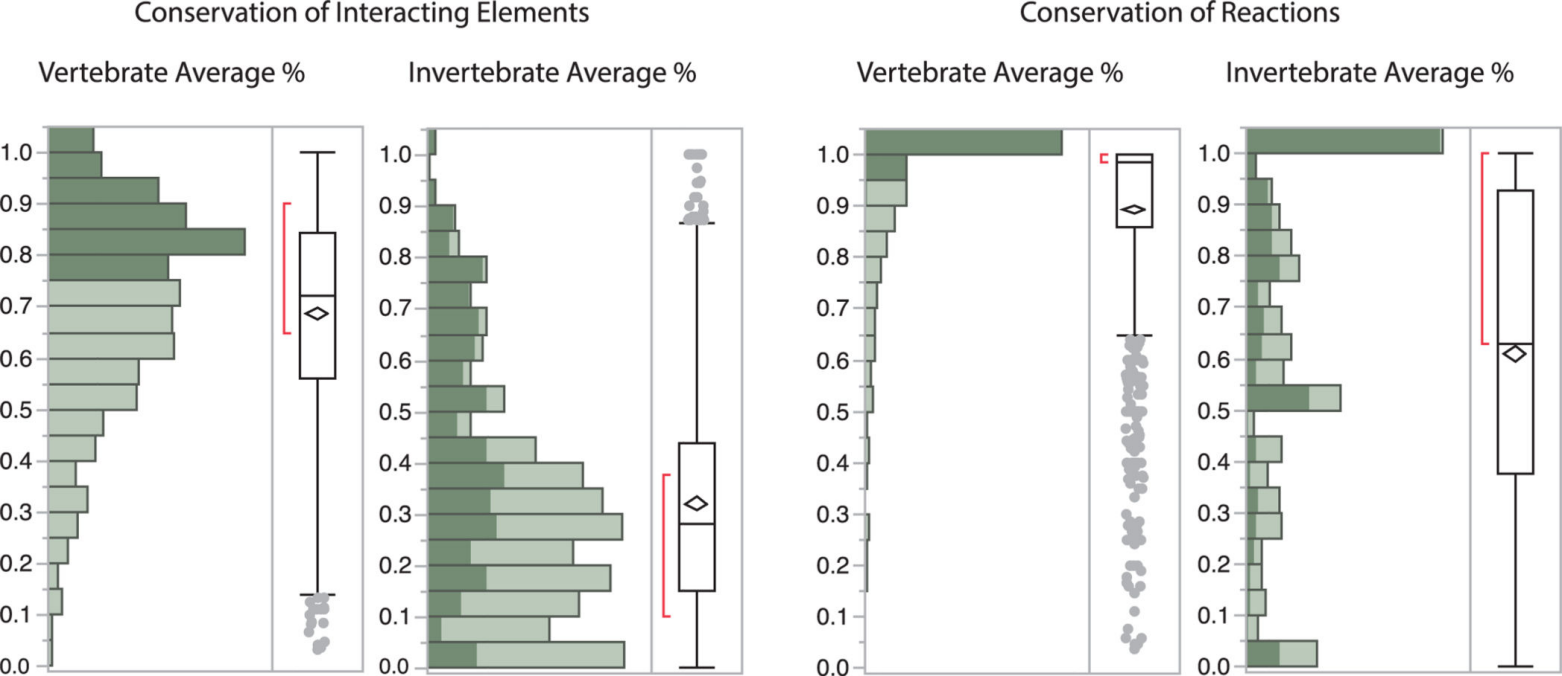
Relative conservation of interacting genes (left panel) and of reactions (right panel) of identified pathways in vertebrates and invertebrates compared to humans. The frequency distribution of pathways consists of percent average numbers among *Mus, Rattus, Danio, Xenopus* and *Gallus* representing the vertebrates, and between *Drosophila* and *Caenorhabditis* representing the invertebrates. The horizontal line within the box plots depicts the median % value, while the diamonds depict the upper and lower 95% of the mean. The ends of the boxes represent the 25^th^ and 75^th^ quantiles, while the brackets outside of the boxes identify the densest 50% of the observations.

**Fig. 4.**
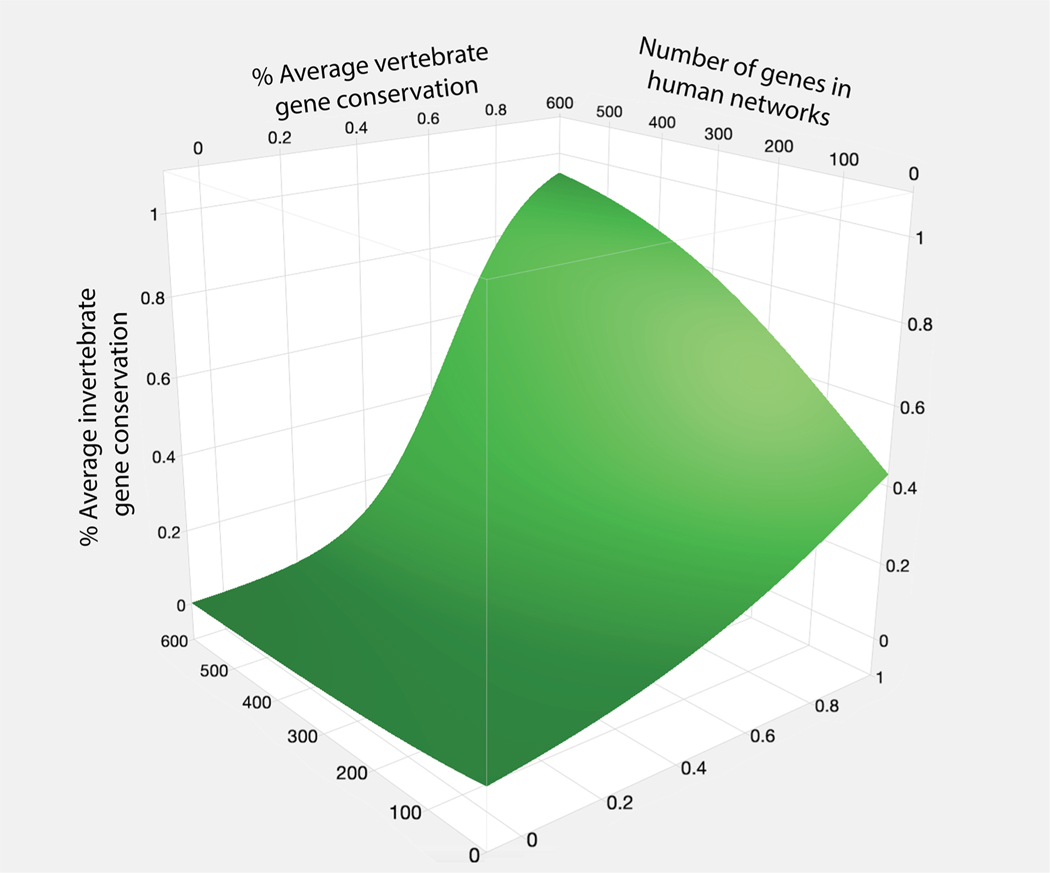
Contour surface plot for 3 factors that predict the likelihood that pathways are conserved between invertebrates and humans, based on the degree of pathway conservation within vertebrates and on the number of interacting genes within the human network. These results are obtained from a generalized linear model (GLM): invertebrate conservation ~ (vertebrate conservation) + (# genes in Homo pathway) + (vertebrate conservation : # genes in Homo pathway).

Pathways, characterized by their *shared genes* among species, are significantly more conserved among vertebrates compared to invertebrates (Fig. 3). On average, vertebrate model species have 69% of the genes that define human pathways. By contrast, *D. melanogaster* and *C. elegans* have only 32% of these pathway genes (36% and 28% respectively). A further investigation of the distribution of the 678 most conserved pathways (containing greater than 75% of pathway genes) among the vertebrates (the shaded fractions in Fig. 3) indicates that only a small fraction (109 or 16%) of these pathways is equally conserved by invertebrates. Nevertheless, the likelihood of conservation for the remaining pathways for invertebrates is significantly associated with the degree of conservation among the vertebrates using a generalized linear model (GLM) (*p* < 0.0001). Moreover, our GLM model also indicates a significant effect of network size on the likelihood of pathway conservation (*p* < 0.0001; Fig. 4). Similarly, pathways as a function of *shared interactions* among species are also significantly more conserved among vertebrates compared to invertebrates (89% *versus* 61% on average; Fig. 3). However, a much larger fraction (323% or 44%) of the 732 pathways that are invariant among the vertebrates are likewise invariant among the invertebrates – while the likelihood of conservation for the remaining pathways for invertebrates is yet more related to the conservation levels of pathways for vertebrates.

Therefore, the prediction of this second test of the comparative approach also holds true. Interactions among genes of human pathways are more strongly conserved among animal species than their individual genes. By consequence, a chemical hazard assessment built upon reactions (i.e., interactions with molecular key events (Sturla et al., 2014)) is likely to be informative for a greater diversity of species.

### 3.3. Condition 3: relative conservation of disease and non-disease pathways

**Fig. 5.**
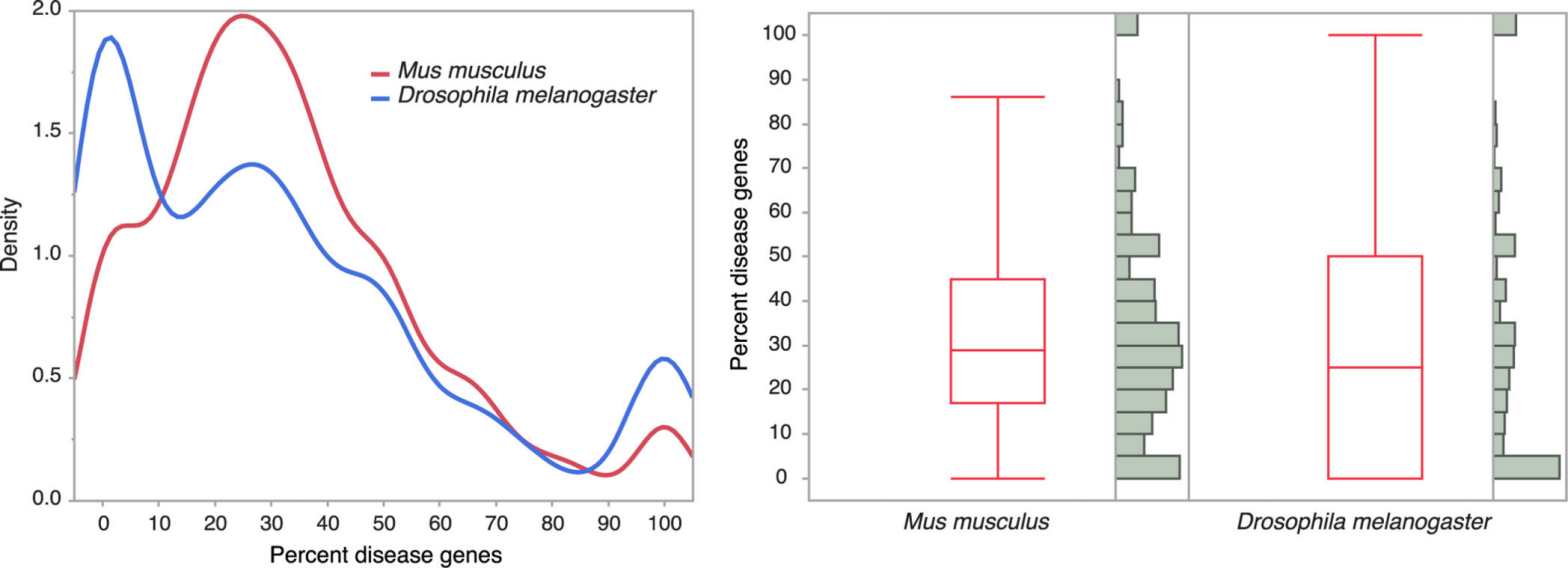
Distribution and composition of homologous gene networks as the percentage of genes that are linked to disease (annotated within the OMIM database), comparing a model vertebrate (*Mus*) and a model invertebrate (*Drosophila*) species. A one-way analysis shows no difference in the mean and distribution of the data between these two species, even though *Drosophila* retains only half the total number of interacting genes within networks compared the *Mus*. Box plots depicts the mean value, while the ends of the boxes represent the 25^th^ and 75^th^ quantiles.

A third test of a comparative approach for chemical hazard assessment is deep conservation in the animal phylogeny of pathways that cause adverse outcomes when perturbed. Following the same reasoning that was applied to discover the earlier evolutionary origins of families containing disease genes, we test whether gene networks that are over-represented by genes linked to disease, or are specifically annotated as disease pathways, are also more ancestral than pathways that have no known links to disease. To evaluate the relative conservation of genes linked to disease within pathways, we recorded the proportion of mapped human orthologs to genes of both *M. musculus* and *D. melanogaster* pathways that are annotated as disease genes within the OMIM database (Supplemental Table A4). Despite the two-fold reduction in the median number of conserved human pathway genes in fly compared to mouse (6 *versus* 12), these two species show no significant difference in the proportion of disease genes across pathways (Fig. 5). This result suggests that disease genes, when investigated in the context of their membership within pathways (instead of loci independent of other genes), have the same likelihood of conservation as non-disease genes for both vertebrates and invertebrates.

**Fig. 6.**
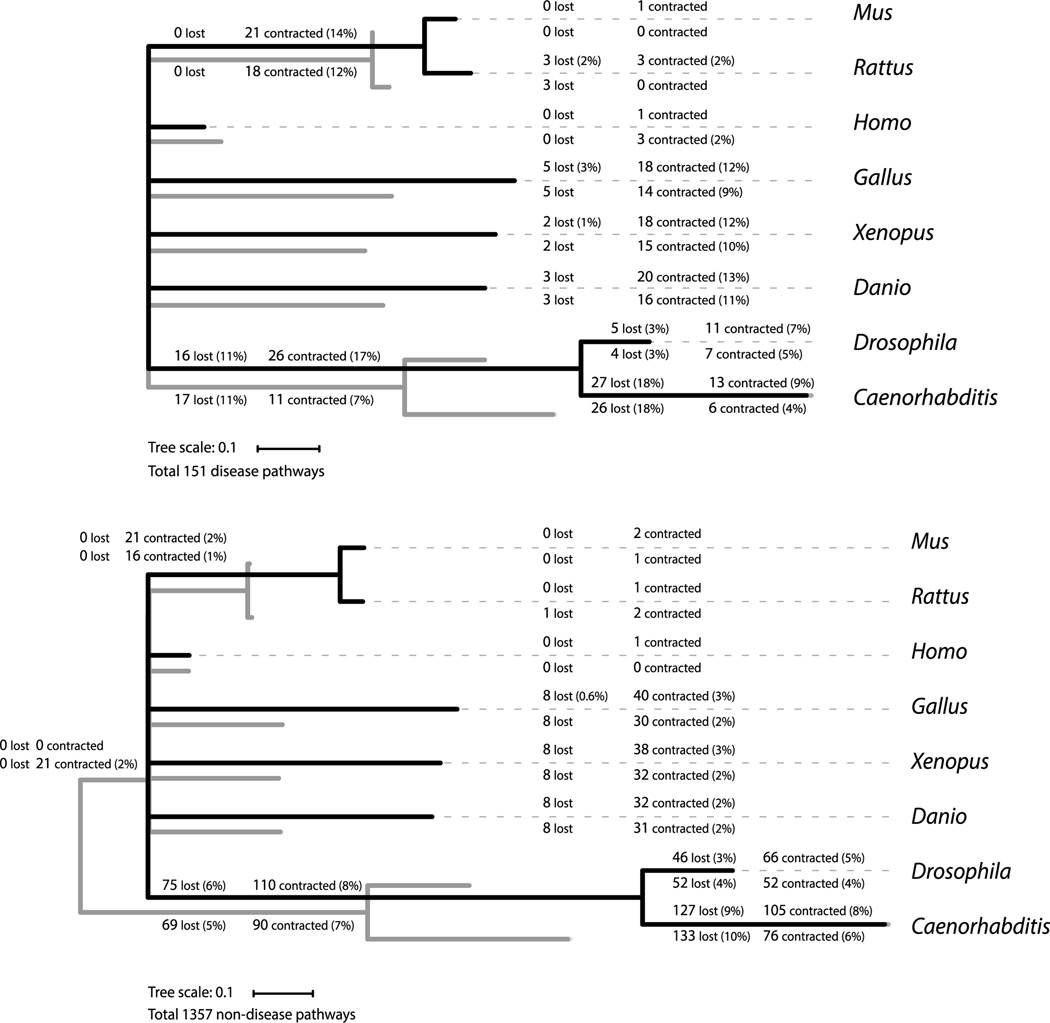
Phylogenetic reconstruction of the contractions and loss of 151 disease (top panel) and 1357 non-disease (bottom panel) pathways by computing likelihoods and posterior probabilities for ancestral network content of conserved genes and reactions among eight species. Branch lengths are proportional to the rates at which genes (black trees) and reactions (grey trees) within networks are lost. A tally of the lineage-specific contractions and loss for both genes (top counts with percentages) and reactions (bottom counts with percentages) are indicated at each branch, which can be summed to obtain the likely total number of lost pathways from the root of the tree to each species. For example, 32% or 31% of disease pathways are lost within invertebrates, while only 18% or 19% of non-disease pathways are lost, based on the number of genes or reaction, respectively.

To specifically compare the conservation of networks (instead of genes) that are annotated as disease and non-disease pathways, a phylogenetic reconstruction of their contraction and loss along the vertebrate and invertebrate branches of the tree was performed, by calculating their posterior distributions at each ancestral node and terminal branches from a probabilistic inference model of only losses (see methods). The results are presented separately for the loss rates of 151 disease pathways and of 1357 non-disease pathways in relation to both the number of homologous genes (Fig. 6, black trees) and their number of homologous reactions forming these networks (Fig. 6, grey trees). An preliminary study of the tree lengths reflecting the branch-specific loss rates indicates (yet again) that the networks of reactions are better conserved among species than the networks defined by their genes; this is observed by the significantly shorter branch distance (by 40–44%) between *Homo* and all other species (trees colored in grey, Fig. 6). To a lesser extent, this result is also reflected by the numbers printed along each branch, indicating the likely number of networks that are lost (by no longer being composed of any genes or reactions) or that are contracted to only a single gene or reaction. Yet by comparing the sum of network loss, whether based on genes or reactions along each branch leading to both *Drosophila* and *Caenorhabditis*, non-disease pathways are found to be more conserved than disease pathways – whereby 18–19% of non-disease networks are lost within the invertebrate lineage, compared to 31–32% of disease networks – most likely because the non-disease networks are on average larger than disease networks (mean difference of 13.7 genes; t ratio = 5.77; *p* < 0.001), thereby consistent with our earlier finding of the size-associated conditions that increase the likelihood of conservation (Fig. 4).

Therefore, the prediction from this third test does not hold true. Disease associations at the level of networks have no detectable impact on the likely conservation of pathways between humans and other animal species. This suggests that networks are generally conserved by the functional dependencies among their interacting genes, irrespective of the health impact of individual disease associations when assessed independently. As a consequence, network functions are most likely better conserved among distantly related species than gene functions for biological read-across.

### 3.4. Condition 4: conservation of hallmark signatures of potential adverse outcome pathways

A final and altogether independent test of a comparative approach for chemical hazard assessment is a gene set enrichment analysis, for gene sets that are hallmarks (i.e., biomarkers) of specific biological states or processes among the top conserved gene families shared by humans and up to 11 model species. These gene sets vary in size between 19 and 132 gene families (Supplemental Table A5) and are grouped together by their involvement in the same pathway, often associated with disease. Our primary interest is to investigate the feasibility of understanding the mechanisms of toxicity across the animal phylogeny, spanning from invertebrates to humans based on a significant number of conserved genes identified from a small number of model species. We therefore test whether a ranking of gene families by the Shannon index, which measures the distribution of genes among the species (see methods), groups highly conserved genes together by their involvement in the same biological pathways. In other words, if the hallmark gene sets that are biomarkers of an adverse outcome fall within the top-ranked list of gene families, such biomarkers are potentially indicative of a chemical hazard for a greater variety of species that can equally serve as human surrogates for understanding toxicity.

**Fig. 7.**
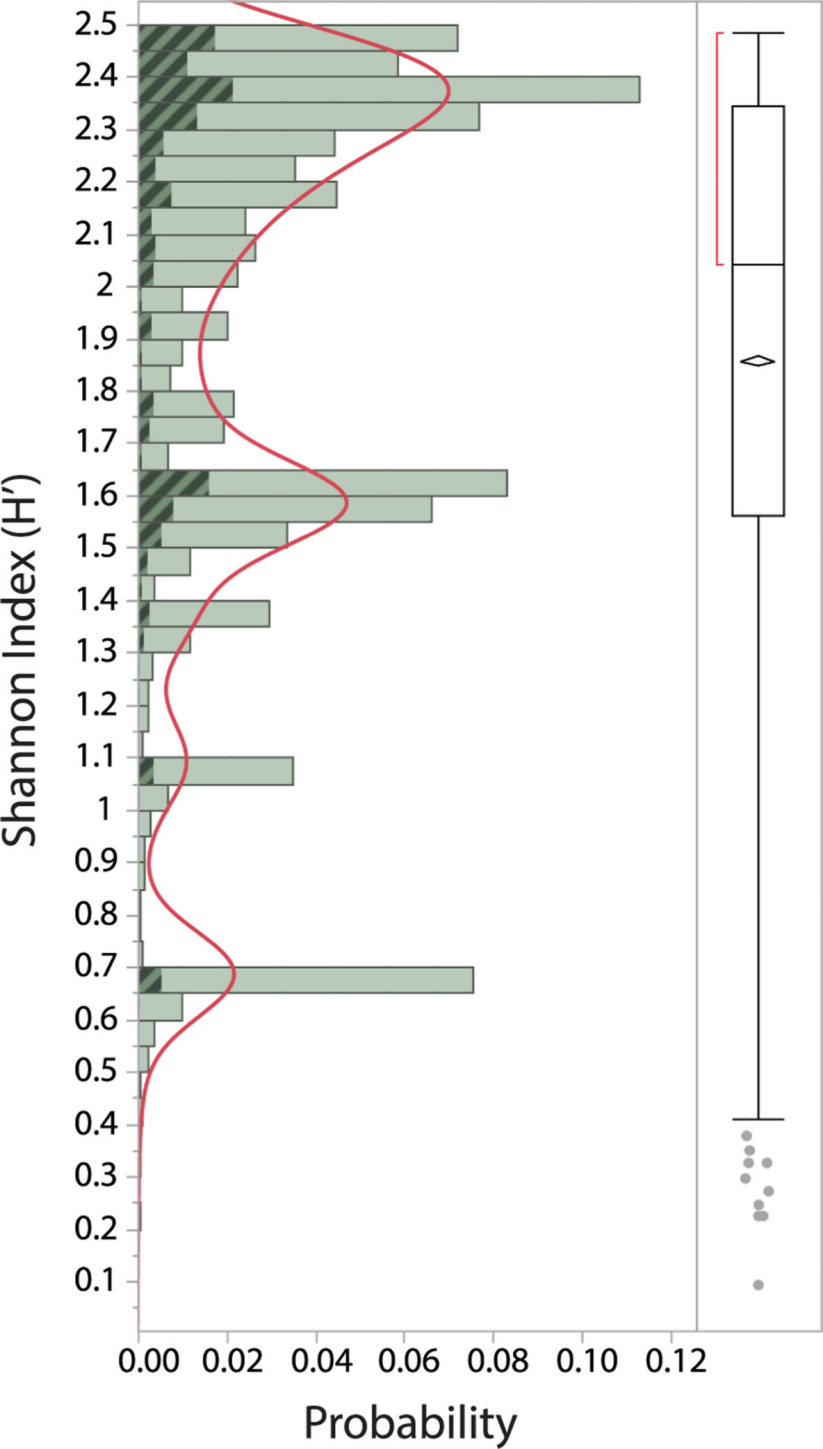
Frequency distribution of gene families based on their Shannon Index (H’). A non-parametric density curve highlights four broadly defined classes of genes. The fraction of disease-associated gene families are shaded. The horizontal line within the box plot depicts the median index value, while the diamond depicts the upper and lower 95% of the mean. The ends of the box represent the 25^th^ and 75^th^ quantiles, while the brackets outside of the box identify the densest 50% of the observations.

The Shannon index (H’) is a popular measure of biodiversity used in ecological studies (Spellerberg and Fedor, 2003) being based on the weighted geometric mean of the proportional abundances of species being equally distributed, typically among different habitats (Keylock, 2005). Here, we apply this index to rank order gene families, based on the relative evenness of their distribution among the genomes of 12 animal species (Suite 1; Supplemental Table A6). A bivariate fit of H’ by the number of species sharing homologous genes demonstrates a high correlation (95%) between the scale of the index and how gene families are partitioned among the species (Supplemental Fig. A2). Indeed, the H’ intervals are narrowly delineating gene families based on species numbers (Supplemental Fig. A2). Overall, the gene families are broadly distributed by their H’ indices into four classes, each loosely defined by their occurrences within distinct phyla. For example, the first and largest class contains homologous genes that are shared by both vertebrates and invertebrates, whereas the second and next largest class contains homologous genes that are mostly restricted to the vertebrates (Fig. 7). The last two classes approximately define gene families that are restricted to tetrapods and finally to mammals. The overall index mean is high (avg = 1.86, std = 0.56), given that H’ for the densest half of the 10,441 gene families is above 2.1 (see red bracket in Fig. 7). This analysis, therefore, indicates that the largest fraction of gene families within the human genome are conserved between vertebrates and invertebrates (H’ > mean).

**Fig. 8.**
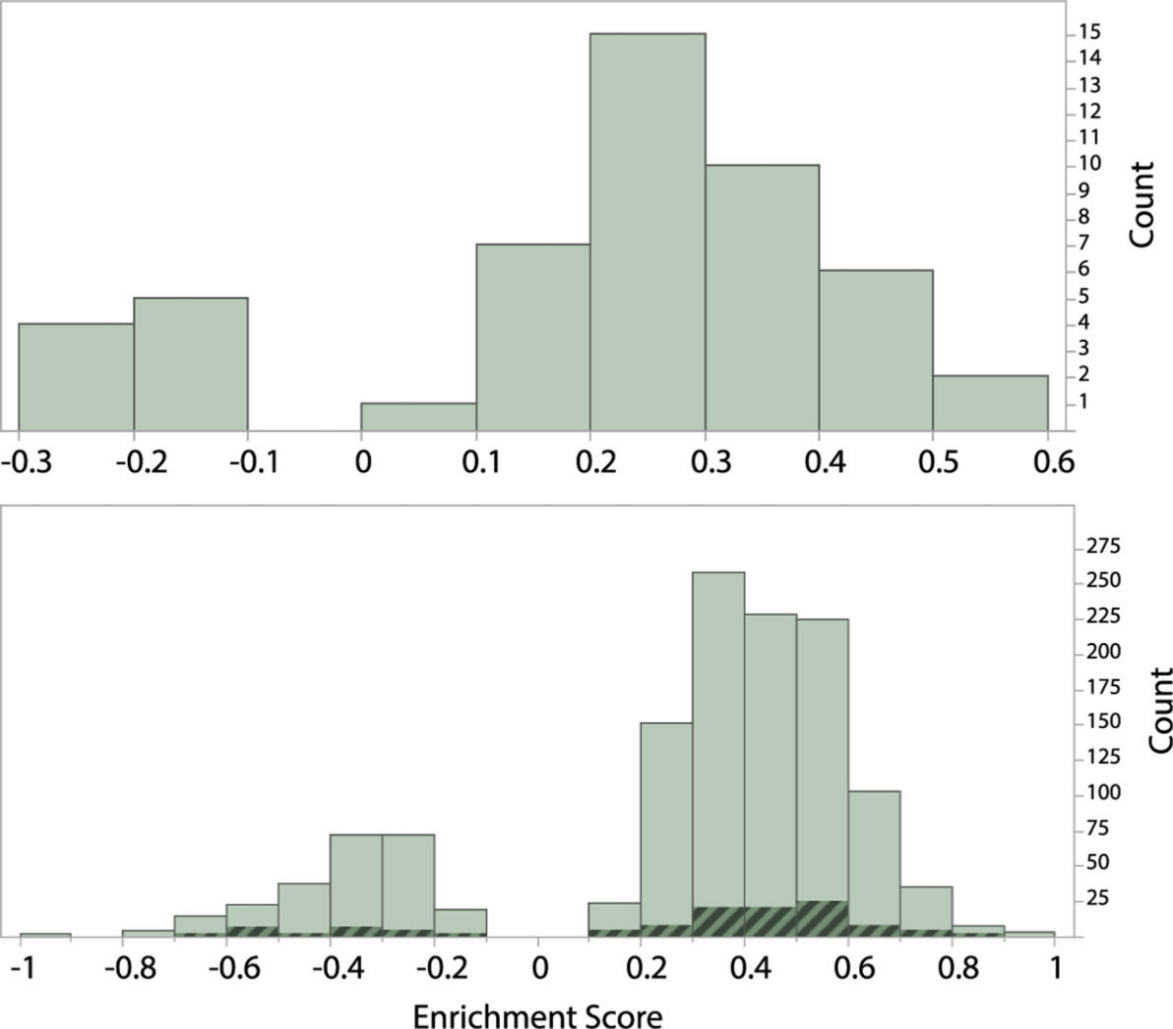
Frequency distributions of gene sets based on their enrichment score from two separate gene set enrichment analyses (GSEA) against a list of 10,441 gene families ranked by their Shannon Index (H’). The first analysis uses 50 Hallmark gene sets obtained from MSigDB (top panel). The second analysis uses 1266 gene sets that consist of Reactome pathways (Supplemental Tables A5–A8). The shaded fraction highlights the 104 disease-associated pathways among the 1266 gene sets.

A gene set enrichment analysis is applied to test whether existing known molecular signatures of biological states or processes (biomarkers) are expected to be useful in hazard assessment when applied to experimental organisms that are distantly related to humans. This analysis tests whether most of the genes within each set fall at either the top or bottom of our list of gene families, which are ranked by their H’ indices. This test was done independently for 50 hallmark gene sets curated at MSigDB (Supplemental Table A5) and for 1266 gene sets that consist of Reactome pathways (Supplemental Table A7). Both the hallmark and Reactome gene sets were first converted to ortholog sets. Both tests produced similar results (Fig. 8), indicating that 82% of the gene sets have positive enrichment scores. The genes comprising 22 (of 41; 53%) and 291 (of 1028; 28%) of these sets are significantly enriched in the most highly conserved gene families (top ranked H’ indices) at the false discovery rate of 5% (Supplemental Table A8). An initial study of these gene sets indicates their involvement in some of the most fundamental biochemical and cellular processes that are common to all eukaryotes. These include cellular biosynthesis, respiration, and the regulation of the cell cycle; DNA replication, repair and recombination; RNA processing; the regulation of metabolic processes, among others that are also implicated with cancer (Supplemental Table A8). For example, a hallmark proto-oncogene set (MYC) is the most highly enriched for deeply conserved gene families. By contrast, only 18% of the gene sets have negative enrichment scores, indicating their involvement in processes that are taxonomically restricted – especially to mammals – of which 3 and 15 gene sets significantly fall among the least conserved gene families (bottom ranked H’ indices) at the false discovery rate of 5%. Their functions are primarily immunoregulatory (including innate and adaptive immunity, inflammation, defense responses), cell communication and signal transduction. For example, the olfactory signalling Reactome pathway gene set is found to be the most taxonomically limited.

**Fig. 9.**
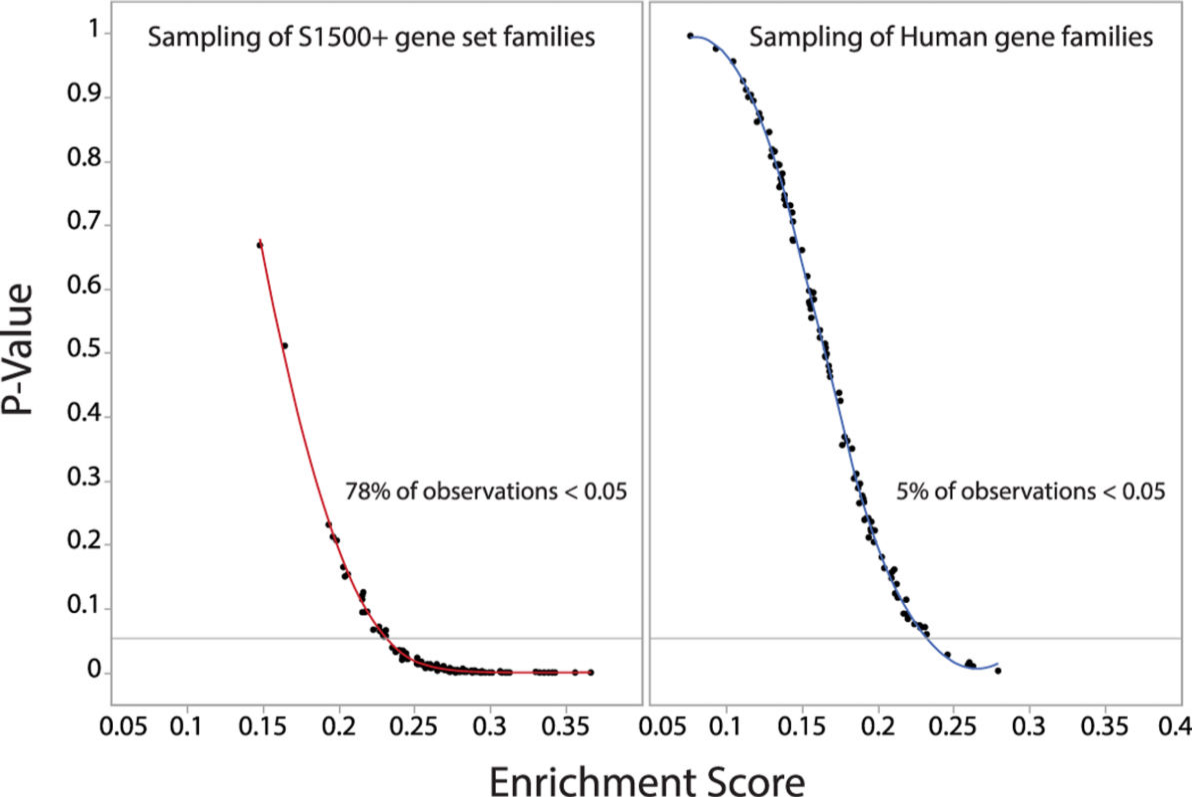
Bivariate plots of the significance levels (p-value) of enrichment scores for 100 gene sets randomly sampled from the US National Toxicology Program’s s1500+ panel of sentinel human genes designed for high-throughput toxicological screening of chemicals by gene expression monitoring (left panel), compared to a random sampling of gene families from the human genome. These results are obtained from a gene set enrichment analysis (GSEA) against a list of 10,441 gene families ranked by their Shannon Index (H’). Seventy-eight percent (78%) of observations for s1500+ gene sets have *p*-values below 5%. By contrast, only 5% of observations for subsamples of the human genome are significantly enriched by phylogenetically conserved gene families (Supplemental Tables A11–A12).

Overall, the conservation of hallmark signatures important to health is sufficiently great to warrant a serious examination into their uses in regulatory toxicology. Importantly, this method can be applied to any *a priori-defined* gene sets that are relevant to toxicology. For instance, the United States National Toxicology Program (NTP) recently published a sentinel set of genes (s1500+ panel) that is designed to best represent biological diversity, address co-expression relationships, and represent known pathways (Mav et al., 2018). A rapid investigation from a random sampling of this gene set against our ranking of gene families demonstrates that the cross-species extrapolation potential of screening results obtained from NTP’s chosen gene set is substantially greater than a random sampling of all gene families from the human genome (Fig. 9).

Therefore, the prediction of this final test of the comparative approach also holds true. Gene sets that already serve as biomarkers of specific biological states or processes, which include stress-response pathways useful for hazard detection in toxicology, are enriched by top-ranked genes based on their evolutionary conservation across the animal phylogeny.

## 4. Discussion

Modern evolutionary thinking in medicine has deeper roots than in toxicology (Nesse and Williams, 1994). As a result, evolutionary medicine is applied today by health care professionals who advance actions to prevent disease; having a better understanding of the causes of ill-health using an evolutionary approach helps people to stay healthier (Benton et al., 2021; Gluckman et al., 2011). It is well known that disease phenotypes, including cancer and developmental defects, are the result of gene-by-environment (G x E) interactions that were essentially shaped by our distant past (Mbemi et al., 2020). For example, many gene regulatory networks in stress response to environmental conditions are ancient. Yet they are highly interconnected to developmental pathways throughout the course of animal evolution, leading to exposure-related defects of human organs via a molecular toxicological response that is shared among all animals (Leung et al., 2017). This present study attempts to further support the practice of safeguarding both environmental and people’s health, by exploring conditions that permit such ‘biological read-across’ in chemical hazard assessment – from human surrogates to ecological sentinels and *vice versa* – based on the use of homology for a more profound understanding of the original causes of exposure-related harm.

We chose to limit our comparative investigation to a modest number of biomedical and ecological model species that have comprehensive genome annotations and that have reliable and pre-computed gene homology assignments in a well-maintained public online database. Numerous other animal models with well-annotated genomes are equally informative of the evolutionary conservation of toxicity-relevant genes and pathways, including other invertebrate species such *Tribolium,* which is enriched with P450 and other detoxification enzymes (Richards et al., 2008). Because toxicology traditionally makes use of a larger array of test species, which include closely related alternative genomic model species (e.g., rat instead of mouse) as well as many species that lack sufficient genomic resources, we provide a reference species tree (Supplemental Fig. A3) that depicts their phylogenetic position among those species used in our study.

Four conditions were tested to build a case for toxicity by descent and support the foundation of a comparative approach for chemical hazard assessment. Our first test demonstrated that known genes of the human genome associated with disease have evolved comparatively earlier than genes unassociated with disease during animal evolution. Although this finding is not new (Forslund et al., 2011), ours is from testing this condition at many branches of the evolutionary tree, to reveal their origins (and losses) within key animal groups with a focus on comparing the vertebrate to invertebrate lineages. For instance, 71% of disease gene families evolved in ancestors of both vertebrates and invertebrates, compared to 59% of the non-disease gene families. Although many of the remaining human gene families, for both classes, originate in the vertebrate ancestor, we found that mammalian-specific genes are disproportionately *unassociated* with disease (76%). Also given that disease-associated gene families are 50% more likely to be retained by the other vertebrates, arguments that rodents (or other mammals) are vastly superior models to understand the human condition are diminished.

Certainly, invertebrates are even more distantly related to humans. Yet, despite the common ancestry of arthropods dating back to a time before the Cambrian period, when most major animal phyla appeared in the fossil record, our study confirms that the crustaceans retain a greater number of ancestral gene families that are shared with humans than insects, irrespective of varying numbers of duplicated genes (Colbourne et al., 2011; Thomas et al., 2020). Furthermore, by comparison to the patterns observed in vertebrate genomes, disease-associated gene families are no more conserved than gene families unassociated with disease, thereby pointing to a general rate difference among major invertebrate lineages in their loss of ancestral gene families, irrespective of disease class. Natural selection on the maintenance of this class of genes is therefore presumably more relaxed among invertebrates because, although they share ancestry with genes causing disease in humans, they are less likely to have the same disease outcomes in these more distantly related species, even though they are likely to retain functional similarities (Leung et al., 2017). If correct, the proposed interpretation of these results suggests that the chemical perturbation of genes in distantly related species may be indicative of their malfunction in processes that are shared with humans, yet not necessarily indicative of the specific adverse outcome to these tests species. Certainly, the differences in the physiology, organ systems, exposures, and life history among organisms would contribute to differences in such outcomes.

To establish a stronger conservation link with processes (instead of genes), our second test demonstrated that reactions within functional pathways are better conserved among distantly related species than the genes that are members of these pathways. For example, 44% of pathways are invariably conserved between vertebrates and invertebrates for their shared reactions, compared to only 16% at sharing at least 3/4 of their genes. This finding suggests that the functional conservation of pathways depends upon only a small fraction of the interacting genes for any given species, including humans. On average, vertebrates are shown to retain 71% of the genes that define human pathways, while invertebrates are shown to retain only 34% of these genes. This finding does not imply that human pathways contain more interacting genes than homologous pathways in other species; our investigation is restricted to genes from each species that are shared with humans. In other words, we intentionally ignored genes that originate within the genomes of lineages that are distinctly non-human (i.e., lineage-specific genes). For example, networks that are shared between humans and an invertebrate species can have equivalent numbers of interacting genes. Yet if so, only 34% are orthologs on average, while the remaining 66% of genes originate in descendants that are specific to either humans or invertebrates. However, our study finds a great amount of variation in that fraction that is shared between humans and a distantly related species, which is most conveniently explained by the number of genes within the human network.

An earlier experimental comparison of interacting genes of the human, *Drosophila* and *Caenorhabditis* genomes showed that the likelihood of identifying homologous gene networks among distantly related species increases by virtue of the network’s size (Gerstein et al., 2014). This discovery is the basis of a helpful model that can now be applied to reducing the use of mammalian species for toxicity testing, by predicting the level of certainty in the cross-species extrapolation of a chemical perturbation to a biomolecular process. A generalized linear model (GLM) constructed from curated networks within the Reactome database permits hazard assessors to make an *a priori* determination on whether gene expression monitoring – for a specific disease or toxicological pathway using an invertebrate – may be indicative of harm to humans. As an example, for large pathways consisting of over 500 genes, vertebrate and invertebrate test species have equal likelihoods of conserving genes of the human pathway. These likelihoods of sharing human orthologs are sharply reduced for pathways consisting of only a dozen genes, where vertebrates are likely to retain more than twice the number of human pathway genes than invertebrates. The reliability of this model requires empirical data showing that homologous genes from vertebrate and invertebrate test species are more similarly co-regulated in response to chemicals when interacting in larger networks. Such coregulatory responses are unexpected for genes that evolved performing independent processes under the same environmental conditions. Although the magnitude of their coregulatory responses, or the dose at which a response is initiated, are known to differ among species (such genetic variation naturally exists, even among populations of the same species (Oleksiak et al., 2002; Whitehead et al., 2010)), this model may be useful for prioritizing disease and toxicity pathways that can be demonstrated in invertebrates and thereby provide supporting information for understanding environmental health.

For example, the European Chemicals Agency (ECHA) and various industries are pursuing methods to improve certainty in the government-mandated hazard assessments of chemicals that are manufactured or imported into Europe, while reducing the use of vertebrate test species (Williams et al., 2009). Additionally, aligning with this global transition to greater use of animal alternatives in chemical safety evaluations, in 2019, the administrator of the US EPA signed a directive that calls for the Agency to eliminate all mammalian study requests and funding by 2035 (Grimm, 2019). Due to these types of regulatory changes, there are efforts to explore the utility of alternatives to animal testing. Pathway-based approaches that capitalize on computational, high-throughput and omics technologies are recommended as methods that can collectively be used to explore cross-species sensitivities to chemicals (Krewski et al., 2010). However, there is relatively limited attention directed at understanding the utility of existing mechanistic toxicology data on non-vertebrate species, despite the pioneering research using invertebrate model species to reveal important aspects of human toxicology (e.g., Devineni and Heberlein, 2013). For instance, alcohol represents a cultivated toxic substance in the human sphere yet is part of the natural environmental toxicology for many non-human species, such as the larvae of many insect species dieting on decaying fruit. Ancestral links to toxic substances permit experimental work using *Drosophila* to identify the genetic basis of human susceptibility to their adverse effects (Morozova et al., 2009). This comparative approach demonstrates the conservation of biology among species as diverse as mammals and insects, providing evidence toward the potential usefulness of invertebrates to inform and identify chemical perturbations that could lead to adverse outcomes in human health.

The chemicals of greatest concern to humans are those that cause cancers, DNA damage, reproductive abnormalities, endocrine disruption, and neurotoxicity. Of 4371 genes that we extracted from MSigDB Hallmark gene sets, 792 have known biomarker applications for one or several of these health outcomes (Supplemental Fig. A4). By interrogating the Ingenuity Pathway Analysis commercial database (IPA, Qiagen, (Kramer et al., 2014)) to retrieve all annotated biomarkers for these five adverse health outcomes, these biomarkers assemble into 437 pathways (*p*-value < 0.05) based on their known functional interactions. As expected, the number of genes within these pathways ranges from many (e.g., 488 genes in the axonal guidance signalling pathway) to very few genes (i.e., two interacting proteins) (Supplemental Table A9). Our results thus enable prioritization of such pathways, beginning with the largest pathway (axonal guidance signalling pathway), which is associated with adverse neurological and cancer outcomes.

Pathway-based approaches are gaining acceptance for grouping and prioritizing data-poor chemicals for more detailed toxicity studies, based on profiling genes that are indicative of the chemical mechanisms of action. For example, the knowledge of toxicity mechanisms for hundreds of per- and polyfluoroalkyl substances (PFAS) is growing to include pathways that are inferred using the Reactome database (Houck et al., 2021; Massarsky et al., 2022). Several pathways identified through this ToxCast-Reactome framework are supported by epidemiology or laboratory studies that signal mechanisms contributing to *(i)* hepatotoxicity via the dysregulation of lipid metabolism by peroxisome proliferator-activated receptor-α (PPAR-α), *(ii)* immunotoxicity via the Fc epsilon receptor and interleukins (ILs) signalling pathways, and *(iii)* disease via the PI3K/AKT signalling in cancer and CDK5 triggered neurodegeneration (see references within Massarsky et al., 2022). Our method of evaluating the likely conservation of pathways – with results presented within the supplemental tables – demonstrates that some, but not all such toxicologically-relevant pathways are expected to be shared between humans and distantly related species when perturbed by PFAS. The GSEA indicates that immunotoxicity by the disruption of interleukins signalling and the aberrant P13K signalling in cancer is likely restricted to tetrapods or mammals. By contrast, the neurogenerative and lipid metabolism pathways of PFAS toxicity are likely to be more broadly conserved to even include the invertebrates. Such predictions are currently being tested (see http://precisiontox.org).

Our third test revealed additional useful properties of pathways, by failing to find (contrary to before) a greater rate of conservation for disease-associated genes (and networks) compared to those unassociated with disease. First, the proportion of disease genes in the defined human networks is found to be equal between mouse and fly, despite disease genes having twice the rate of conservation compared to non-disease genes in mouse, but not in fly when genes are appraised independently of their memberships within pathways. Therefore, the outcomes of natural selection are presumably different for networks, shaped by the epistatic interactions among the genes for their combined effects on the organism’s fitness. Second, a statistical model of the rates at which reactions are lost along the branches of the animal phylogeny once again demonstrates that networks defined by their reactions are better conserved than those defined by their homologous genes. Yet, no rate differences are observed between disease and non-disease-associated networks.

Epistasis in genetics refers to the non-independence of the effects of different genes (Phillips, 2008), which may be indirect when the genes encode components of metabolic, developmental or regulatory networks. For example, the aryl-hydrocarbon receptor (AhR) is a key regulator of enzyme detoxification, by initiating the transcription of the cytochrome P450 gene family that metabolizes toxic dioxins through oxidation reactions (Fujii-Kuriyama and Mimura, 2005). When inactive, AhR forms a complex with Hsp90 plus at least five other proteins in the cytoplasm and serves as a receptor for chemicals including dioxins (Hankinson, 1995). When activated by ligands, AhR dissociates from Hsp90 and dimerizes with the ARNT within the nucleus, serving as a transcription factor for xenobiotic response genes (Mimura and Fujii-Kuriyama, 2003). This AhR signalling pathway requires the coordinated actions of many interacting proteins and co-factors. In such cases, evolutionary theory predicts that when epistatic interactions are strong, natural selection on one genetic element of the pathway usually constrains evolutionary changes on the other functionally dependent elements (Segre et al., 2005). For example, a genome-wide investigation of natural killifish populations that evolved to tolerate normally lethal levels of dioxins revealed signatures of natural selection at a majority of known genetic elements of the AhR pathway (Reid et al., 2016). Therefore, epistatic interactions are the likely reasons for the shared rates of gene conservation within pathways, irrespective of disease class in our study.

This comparative approach thus sets useful provisions for evaluating environmental health hazards of chemicals – using a convenient suite of distantly related (non-mammalian and non-vertebrate) model test species that are relevant to both human- and eco-toxicology (e.g., *Daphnia, Hyallella, Fundulus*) – by monitoring already known biomarkers of disease outcomes that are prioritized by the size of their gene networks (Condition 2) and the conservation rankings of their gene families (Condition 4). Our investigation of the Shannon index (H’) – applied here for ranking gene families within the human genome based on their patterns of evolutionary conservation in other animal species – indicates that 53% of the human hallmark gene sets curated at MSigDB are significantly composed of conserved gene families, many of which include genes with known biomarker applications for the disease outcomes of greatest concern to chemical hazard assessors. For example, xenobiotic metabolism signalling is represented by 94 unique families of genes within the MSigDB hallmark gene set, which is significantly conserved among distantly related species (FDR *q* < 0.01; Supplemental Table A8). Of these, 42 are known biomarkers for DNA damage / cancer (from a search of the IPA database), which themselves are highly conserved (FDR *q* < 0.01; Supplemental Tables A9–A10). Therefore, because these biomarkers are here shown to be members of evolutionarily conserved gene families, and because they also interact in a known large pathway (i.e., 287 genes), these are likely to be most informative of chemical hazards for cancer in humans by monitoring their regulatory responses in model test species. Thus, a conceptual approach to prioritising sets of known molecular biomarkers for their potential utility in screening chemicals for their health hazards considers (1) the genes’ evolutionary origins, (2) their memberships within pathways, and (3) the size of their pathways.

Certainly, not all pathways are deeply conserved, as observed by the negative enrichment scores for genes comprising sets that are known to primarily function within inflammatory and immunological pathways. Yet, while demonstrating limits to the use of molecular biomarkers of human relevance in distantly related species, this result is consistent with those obtained by Maxwell et al. (2014), who had earlier identified distinct classes of human disease genes having more recent evolutionary origins, thereby increasing confidence in the value of the remaining pathways identified in this current study.

The results given here propose accelerating a comparative approach for chemical hazard assessment and advancing cross-species extrapolation of the results. The US Environmental Protection Agency has already developed a web-based tool that begins to incorporate an understanding of molecular similarity in chemical-protein interactions across species to predict chemical susceptibility (LaLone et al., 2016). Phylogenetic toxicology thus has the potential to complement the human-focused NAM developments as part of integrated human health assessment strategies such as those outlined by (Baltazar et al., 2020; Carmichael et al., 2009; Westmoreland et al., 2010). When coupled with pathway-based assessments of toxicity, we anticipate that such methods will expand the paradigm of using distantly related model organisms to provide mechanistic information relevant to human health and environmental safety assessments.

## Supplementary Material

Supplement1

Supplement2

## Figures and Tables

**Table 1:** List of species used for this study forming Suite 1 and Suite 2. The gene numbers and the number of gene families that are shared with humans at the metazoan root of the phylogeny are obtained from OrthoDB release 10.

Species name	Common name	NCBI taxonomic identifier	Suite	Number of genes	Number of gene families	Number of genes in gene families
*Homo sapiens*	Humans	9606	1 & 2	21,933	10,441	18,517
*Mus musculus*	Mouse	10090	1 & 2	22,935	10,282	19,198
*Rattus norvegicus*	Brown rat	10116	2	23,126		
*Xenopus tropicalis*	Clawed frog	8364	1 & 2	21,192	9018	17,709
*Fundulus heteroclitus*	Killifish	8078	1	29,153	8694	20,110
*Gallus gallus*	Chicken	9031	2	30,300		
*Danio rerio*	Zebrafish	7955	1 & 2	29,369	8839	21,597
*Drosophila melanogaster*	Fruit fly	7227	1 & 2	17,049	4683	7324
*Anopheles gambiae*	Mosquito	7165	1	13,035	4589	6846
*Nasonia vitripennis*	Jewel wasp	7425	1	15,593	5204	7660
*Daphnia magna*	Water flea	35525	1	29,112	5248	9693
*Daphnia pulex*	Water flea	6669	1	30,193	5560	10,865
*Hyalella azteca*	Amphipod	294128	1	18,560	5611	9554
*Caenorhabditis elegans*	Roundworm	6239	1 & 2	20,571	4052	6,889

**Table 2:** Distribution of disease, non-disease and mixed gene families among the vertebrate and invertebrate species from Suite 1. Single copy gene families have no duplicates (paralogs) in any studied genome.

Gene family	Disease	Non-Disease	Mixed	Total
Total number	1597	7446	1398	10,441
Single copy	640	3033	0	3673
In all species	414	1327	571	2312
In all species, as single copy	106	324	0	430
In vertebrate & invertebrate	1133	4391	1147	6671
In vertebrate	1596	7430	1398	10,424
In all vertebrates	1379	5380	1349	8108
Only in vertebrate	372	1869	230	2471
Only in all vertebrates	464	3055	251	3770

**Table 3:** The numbers of gains and losses of 1597 disease gene families for each branch of the animal phylogeny compared to the average numbers of non-disease gene families from 1000 random sub-samplings of 1597 of the total number of the non-disease gene families (7446). SD = standard deviation. SE = standard errors of the mean. 99% interval is the upper distribution limit of observations from the mean. Z score is the number of standard deviations of the disease gene numbers away from the mean non-disease gene numbers.

	Disease	Non-disease genes				
	Actual	Average	SD	SE	99% Interval	Z score
Presence *Homo sapiens*	1597	1597.0	0.00	0	1597	
*Mus musculus*	1583	1566.0	4.82	0.15	1566.4	3.53
*Xenopus tropicalis*	1476	1323.7	13.02	0.41	1324.7	11.70
*Fundulus heteroclitus*	1458	1259.4	14.77	0.47	1260.5	13.44
*Danio rerio*	1469	1287.5	13.89	0.44	1288.5	13.07
*Daphnia magna*	917	713.0	16.66	0.53	714.2	12.24
*Daphnia pulex*	963	762.2	17.16	0.54	763.5	11.70
*Anopheles gambiae*	802	625.0	17.04	0.54	626.3	10.39
*Hyalella azteca*	967	771.3	17.54	0.55	772.6	11.16
*Caenorhabditis elegans*	720	534.0	16.49	0.52	535.2	11.28
*Nasonia vitripennis*	887	716.1	18.15	0.57	717.4	9.41
*Drosophila melanogaster*	826	644.1	17.44	0.55	645.4	10.43
Gains Mammalia	59	182.9	11.27	0.36	183.7	−11.00
Tetrapoda	33	71.5	7.26	0.23	72.0	−5.30
Vertebrata	372	401.9	15.44	0.49	403.0	−1.94
Bilateria	1133	940.7	16.92	0.53	941.9	11.37
Losses Fish	12	23.7	4.30	0.14	24.0	−2.72
Cladocera	62	71.6	7.36	0.23	72.1	−1.30
Crustacea	29	35.6	5.36	0.17	36.0	−1.23
Diptera	63	73.6	7.06	0.22	74.1	−1.50
Hexapoda	145	139.8	9.93	0.31	140.5	0.52
Arthropoda	20	16.8	3.54	0.11	17.1	0.90
*Mus musculus*	14	31.0	4.82	0.15	31.4	−3.53
*Xenopus tropicalis*	62	90.4	7.92	0.25	91.0	−3.59
*Fundulus heteroclitus*	35	59.6	6.71	0.21	60.1	−3.67
*Danio rerio*	24	31.5	5.11	0.16	31.9	−1.47
*Daphnia magna*	105	103.8	8.60	0.27	104.4	0.14
*Daphnia pulex*	59	54.6	6.55	0.21	55.1	0.67
*Anopheles gambiae*	103	85.5	7.82	0.25	86.1	2.24
*Hyalella azteca*	117	117.0	8.99	0.28	117.7	0.00
*Caenorhabditis elegans*	413	406.6	15.60	0.49	407.7	0.41
*Nasonia vitripennis*	81	68.0	6.92	0.22	68.5	1.88
*Drosophila melanogaster*	79	66.4	6.99	0.22	66.9	1.80
