# Supplementary material for: Toxicity by descent: A comparative approach for chemical hazard assessment
Source: Environ Adv. Author manuscript; Available in PMC 2024 Sep 3. (PMC11370884; doi:10.1016/j.envadv.2022.100287)
Supplement: Supplement1 [file NIHMS1938539-supplement-Supplement1.pdf]

Supplemental Materials

Figures and Table legends

Toxicity by descent: a comparative approach for chemical hazard assessment

John K. Colbourne[1,2*], Joseph R. Shaw[3], Elena Sostare[1], Claudia Rivetti[4], Romain Derelle[2], Rosemary Barnett[1], Bruno Campos[4], Carlie LaLone[5], Mark Viant[1,2], and Geoff Hodges[4]

[1.] Michabo Health Science Ltd, Coventry CV1 2NT, UK.

[2.] School of Biosciences, University of Birmingham, Edgbaston B15 2TT, UK.

[3.] O'Neill School of Public and Environmental Affairs, Indiana University, Bloomington 47405, USA.

[4.] Safety and Environmental Assurance Centre, Unilever, Colworth Science Park, Sharnbrook MK44 1LQ, UK.

[5.] US Environmental Protection Agency, Duluth 55804, USA.

[*] Corresponding author: john@michabo.co.uk

Figures A1 to A4

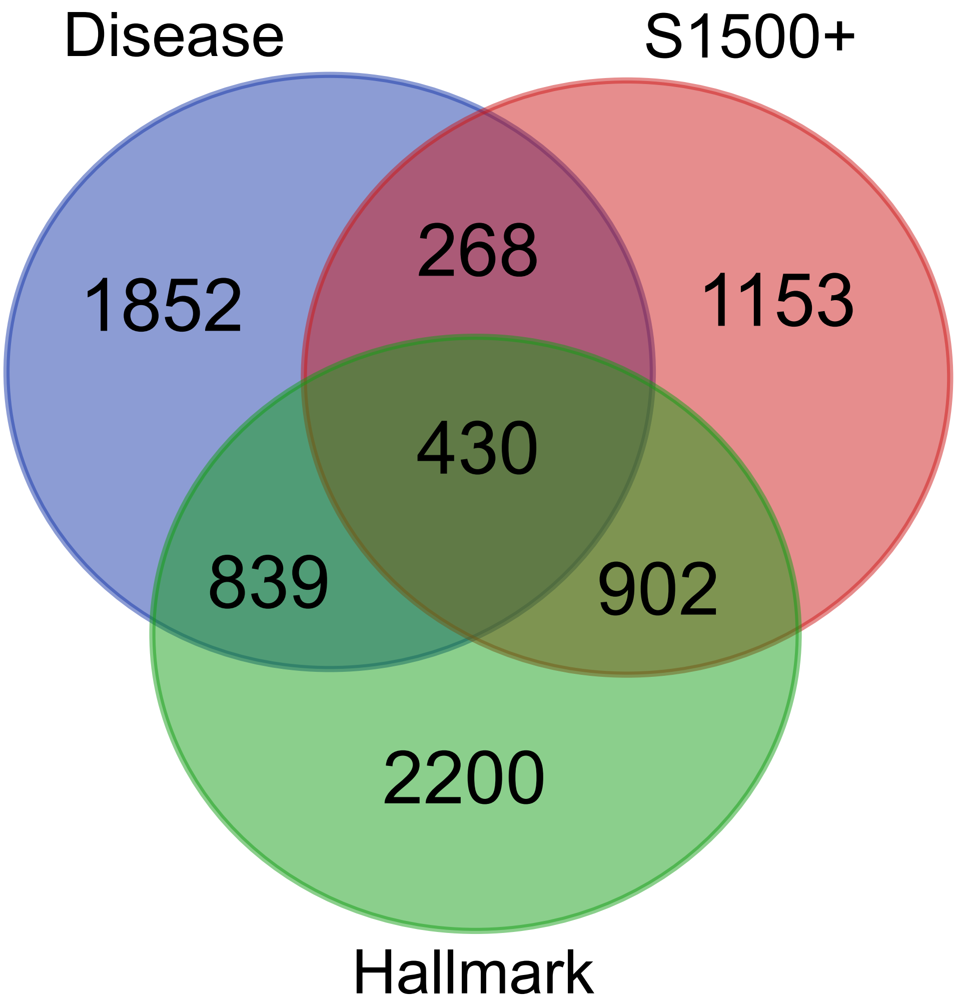

Figure A1. Venn diagram of the distribution of 7644 genes within the MSigDB Hallmark genes sets, the OMIM disease gene set and the National Toxicology Program S1500+ gene set.

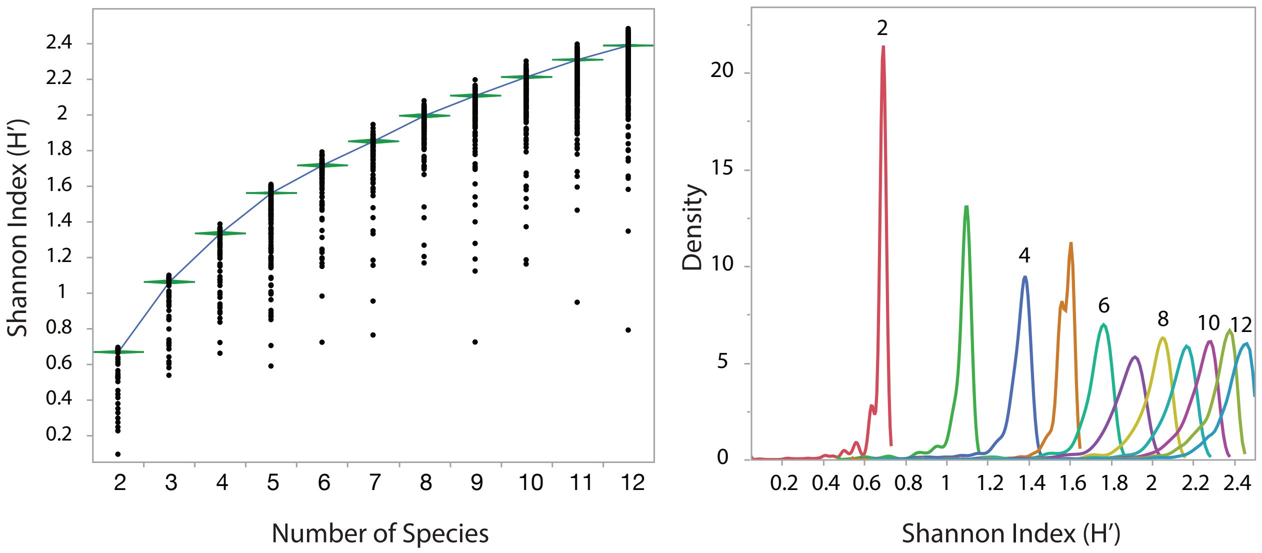

Figure A2. Quality assurance plots indicating the reliability of the Shannon Index (H') at rank ordering 10,441 gene families present in the human genome, based on their relative conservation (evenness of their gene distribution) among the genomes of 12 animal species. A one-way analysis of the H' for all gene families by the number of species (left panel) demonstrates a 95% correlation by virtue of the significant upward shift of the index means (blue line). Diamonds depict the upper and lower 95% of the means. The density plot (right panel) demonstrates clear H' intervals that partition gene families based on the number of species sharing genes by descent. The density unit is the percentage of gene families (dots in the left panel) that fall within the bins assigned to the number of species at all H' intervals.

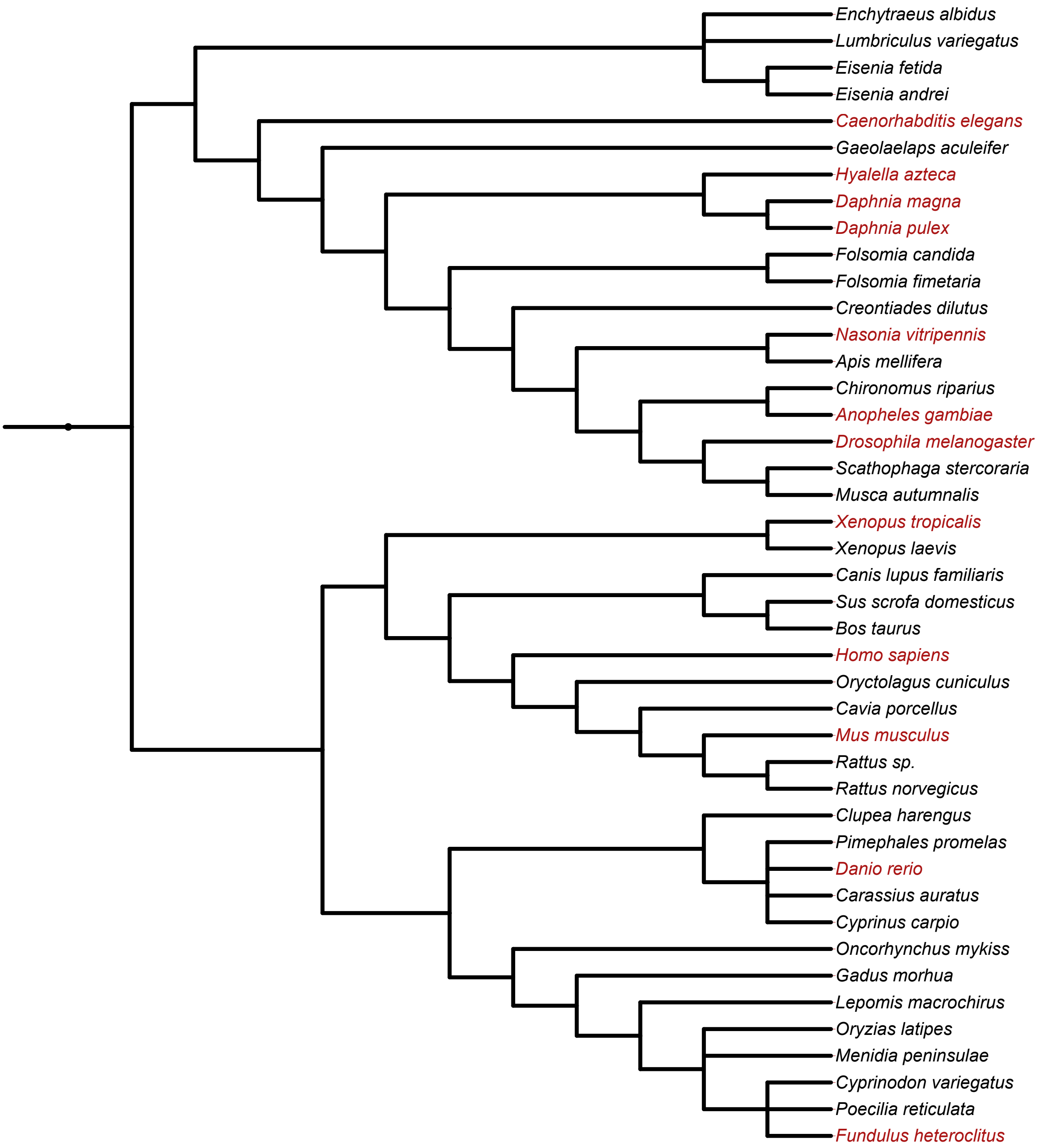

Figure A3. An animal phylogenetic tree containing the species used for this study (*Homo*, *Mus*, *Xenopus, Danio*, *Fundulus, Drosophila*, *Anopheles*, *Nasonia,* two species of *Daphnia*, *Hyalella and Caenorhabditis*) plus OECD recommended species for toxicity testing. Branch structure indicates evolutionary relationships among species. Branch length is not indicative of the time of divergence.

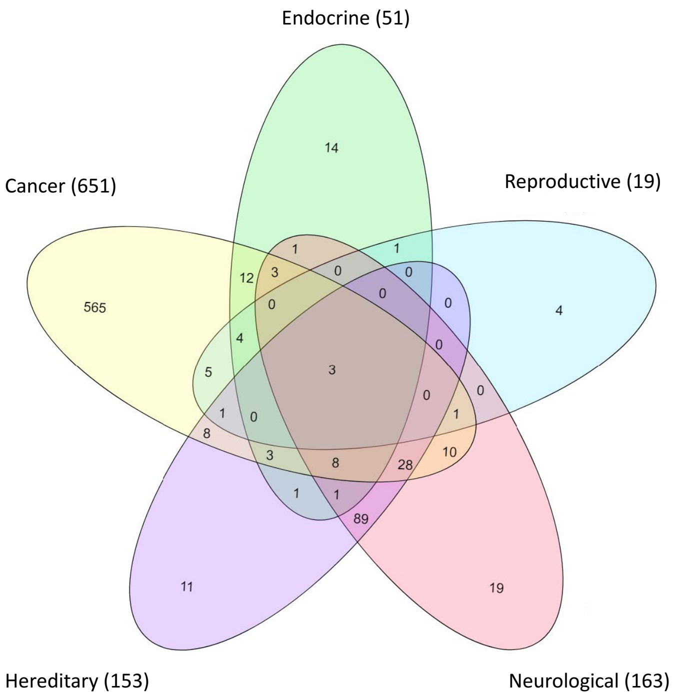

Figure A4. Venn diagram of the distribution of 792 unique and conserved genes within the MSigDB Hallmark genes sets that have known biomarker applications for disease outcomes of greatest concern to chemical hazard assessors: carcinogenic, mutagenic (adverse hereditary), adverse reproductive, adverse endocrine and adverse neurological outcomes. Most biomarker genes are associated with cancer, while adverse reproductive outcomes have the fewest biomarkers. Adverse neurological and hereditary outcomes share a large number of biomarkers (89) and only a few biomarkers are universal for all outcomes.

Table legends

Table A1. List of human genes listed in the OMIM database with their various accession numbers.

Table A2. Number of genes within 12 animal genomes (Suite 1) belonging to gene families present in the human genome. Data were obtained from the OrthoDB database.

Table A3. Number of genes within 12 animal genomes (Suite 1) belonging to gene families that map to 18,810 loci of the human genome, which are annotated as disease, non-disease and unknown based on information obtained from the OMIM database.

Table A4. Number of genes and number of reactions found for 2180 human pathways shared among eight species (Suite 2), including gene identifiers for *Mus* and *Drosophila* and their proportions of disease-associated genes within pathways. Pathways are also annotated as lowest level and disease pathways, obtained from the Reactome pathway database.

Table A5. List of gene families within each of the fifty MSigDB hallmark gene sets.

Table A6. Calculation of the Shannon index (H') for each gene family based on the distribution of orthologs across the 12 (Suite 1) species.

Table A7. List of gene families within each gene set that consists of Reactome pathways. Only gene sets greater than 3 and less than 500 gene families were used in the gene set enrichment analysis.

Table A8. The results of the gene set enrichment analysis (enrichment scores and significance thresholds) for the fifty MSigDB hallmark gene sets and the 1266 gene sets that consist of Reactome pathways measured against the homologous gene sets ranked according to their relative evolutionary conservation by their Shannon indices (H'). Size = number of gene families. ES = enrichment score. NES = normalized enrichment score. NOM p-val = nominal p-value. FDR q-val = false discovery rate. FWER p-val = familywise-error rate. Rank at Max = the position in the ranked list at which the maximum enrichment score occurred.

Table A9. The mapping of biomarkers for cancers, DNA damage, reproductive abnormalities, endocrine disruption and neurotoxicity to 437 human pathways retrieved from the Ingenuity Pathway Analysis (IPA, Qiagen, (Kramer et al. 2014)) commercial database.

Table A10. The results of the gene set enrichment analysis (enrichment scores and significance thresholds) for the 416 gene sets of biomarkers (for cancers, DNA damage, reproductive abnormalities, endocrine disruption and neurotoxicity) identified from the Ingenuity Pathway Analysis (IPA, Qiagen, [39]) commercial database, measured against the homologous gene sets ranked according to their relative evolutionary conservation by their Shannon indices (H'). Size = number of gene families. ES = enrichment score. NES = normalized enrichment score. NOM p-val = nominal p-value. FDR q-val = false discovery rate. FWER p-val = familywise-error rate. Rank at Max = the position in the ranked list at which the maximum enrichment score occurred.

Table A11. List of 100 sets of homolog gene sets representing random subsets of 100 genes from the United States National Toxicology Program's s1500+ panel of markers used in a gene set enrichment analysis.

Table A12. List of 100 sets of homolog gene sets representing random subsets of the human genome used in a gene set enrichment analysis to contrast results against the results obtained from the Program's s1500+ panel of markers.
